# A Salvage Hybrid Reconstruction Using Covered Stents for Internal Iliac Artery Preservation during Endovascular Aneurysm Repair in a Patient with an Extremely Short Common Iliac Artery: A Case Report

**DOI:** 10.3400/avd.cr.25-00149

**Published:** 2026-05-13

**Authors:** Akihito Ohkawa, Kyousuke Miki, Naomi Yasuda, Hitoki Hashiguchi

**Affiliations:** Department of Cardiovascular Surgery, Prefectural Kitami Hospital, Kitami, Hokkaido, Japan

**Keywords:** short common iliac artery, endovascular aneurysm repair (EVAR), salvage procedure, internal iliac artery preservation

## Abstract

The anatomy of the short common iliac artery (CIA) limits distal sealing during endovascular aneurysm repair (EVAR) and often necessitates internal iliac artery (IIA) embolization. We report the case of a 73-year-old man with an infrarenal abdominal aortic aneurysm and an extremely short right CIA that precluded open repair and application of iliac branch devices. As a salvage approach, EVAR was combined with the placement of a covered stent from the right external iliac artery to the IIA and a femorofemoral bypass. Follow-up imaging at 21 months demonstrated preserved pelvic circulation, absence of aneurysm sac enlargement, and sustained graft patency without limb ischemia.

## Introduction

A short common iliac artery (CIA) limits the ability to secure a sufficient distal landing zone in endovascular aneurysm repair (EVAR).^1)^ In such situations, exclusion of the aneurysm often necessitates internal iliac artery (IIA) embolization, which can lead to pelvic ischemic complications, including buttock claudication, sexual dysfunction, and colonic ischemia.^2,3)^ The importance of maintaining pelvic circulation has been emphasized because interruption of IIA blood flow may significantly increase the risk of ischemic morbidity.^4)^ Although iliac branch devices (IBDs) are an ideal solution for preserving IIA perfusion,^5)^ their use may be restricted by anatomical constraints and regional availability, particularly in patients with an extremely short CIA.

We report the case of an infrarenal abdominal aortic aneurysm with an extremely short right CIA, in which standard endovascular options, including IBDs, were not applicable. Therefore, as a salvage hybrid approach, EVAR was combined with the placement of a balloon-expandable covered stent from the right external iliac artery (EIA) to the IIA, with intentional occlusion of the right CIA. In addition, a femorofemoral crossover bypass was performed to maintain right lower-limb perfusion. This case illustrates a feasible alternative strategy for preserving pelvic circulation in carefully selected patients with a challenging iliac anatomy.

## Case Report

**Fig. 1:**
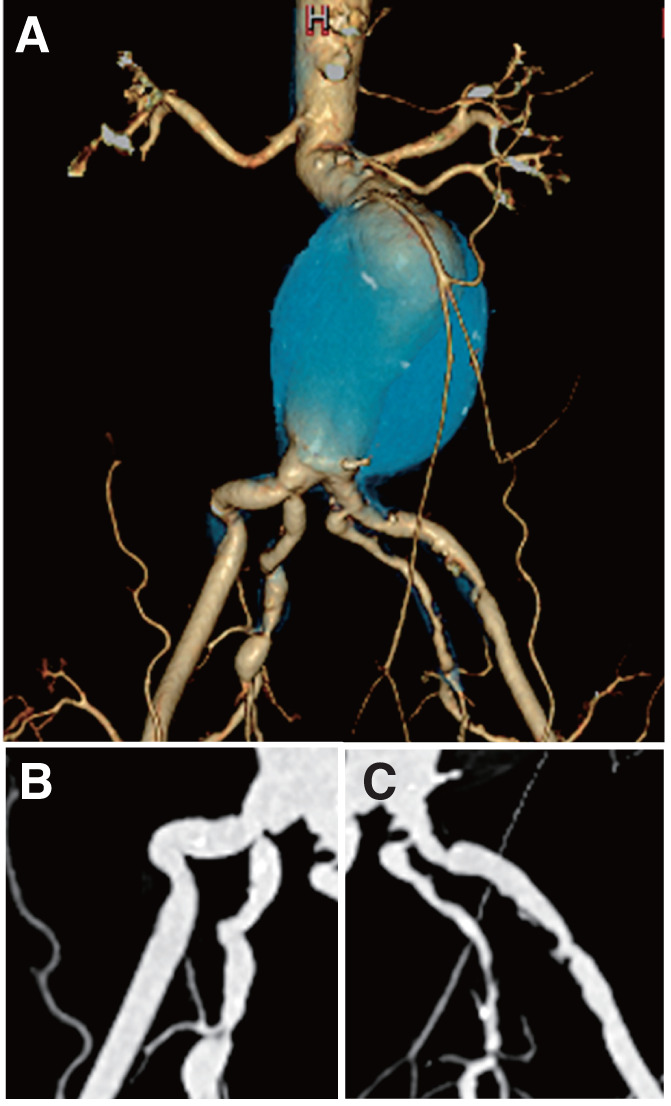
Preoperative computed tomography findings. (**A**) Three-dimensional reconstructed image showing the extremely short right common iliac artery. (**B**) Longitudinal view of the right iliac artery showing a short and narrow right common iliac artery (length, 9.6 mm; diameter, 10–12 mm) with severe ostial stenosis of the internal iliac artery (3.2 mm). (**C**) Longitudinal view of the left iliac artery demonstrating marked hypoplasia and severe ostial stenosis of the internal iliac artery (2.3 mm). These anatomical features precluded the use of iliac branch devices.

A 73-year-old man with a history of cerebral infarction resulting in left hemiparesis, hypertension, and diabetes mellitus was referred for treatment of an infrarenal abdominal aortic aneurysm. Preoperative computed tomography (CT) revealed an extremely short right CIA (length, 9.6 mm; diameter, 10–12 mm), making standard distal landing impossible. The right IIA was patent but showed severe ostial stenosis (minimum diameter, 3.2 mm), whereas the left IIA had marked hypoplasia with severe ostial stenosis (diameter, 2.3 mm) (**Fig. 1**).

Given his neurological status and functional limitations owing to a prior cerebral infarction, the patient was considered unsuitable for open surgical repair. Based on the preoperative anatomical and clinical findings, standard endovascular options were also considered unsuitable. The extremely short and narrow right CIA, together with severe ostial stenosis of both IIAs, rendered the use of IBDs anatomically unfeasible. In particular, the left IIA ostium measured 2.3 mm in diameter, indicating marked hypoplasia; therefore, preservation of the left IIA was considered unreliable, and the left EIA was preferentially selected as the distal landing zone.

A salvage hybrid strategy was adopted to achieve complete aneurysm exclusion while utilizing the narrow right CIA. This involved the placement of a covered stent from the right EIA to the right IIA, allowing intentional occlusion of the right CIA and elimination of inflow from the CIA into the aneurysm sac. Furthermore, an aorto-uni-iliac (AUI) configuration with distal landing in the left EIA was selected to achieve secure aneurysm exclusion. Thus, EVAR was performed using an Endurant AUI device (Medtronic, Minneapolis, MN, USA).

Both common femoral arteries (CFAs) were exposed via bilateral groin cut-downs. An 8-Fr introducer sheath (Terumo, Tokyo, Japan) was inserted through the right CFA, and a 0.035-inch Radifocus guidewire (Terumo) with a KMP catheter (Merit Medical Systems, South Jordan, UT, USA) was advanced into the superior gluteal artery to secure access to the right IIA. The guidewire was then replaced by a stiff Radifocus guidewire. A 12-Fr Dry Seal sheath (W. L. Gore & Associates, Flagstaff, AZ, USA) was advanced from the right CFA into the right IIA. A VIABAHN covered stent (13 × 100 mm) was deployed from the right IIA to the right EIA to bridge the stenotic ostium, followed by balloon touch-up to ensure adequate expansion. Subsequently, an 8-Fr introducer sheath was inserted through the left CFA. A pigtail catheter was advanced to the distal subclavian level, and a stiff guidewire was positioned. The AUI device was deployed from just below the renal arteries to the left CIA and extended into the left EIA using an additional limb (ETLW1610C124EJ) to secure an adequate distal landing zone. The left IIA was intentionally occluded. Completion angiography demonstrated only a type IV endoleak, with no evidence of type I or type III endoleaks.

Because the right CIA occlusion eliminated antegrade flow to the right lower limb, a femorofemoral crossover bypass was constructed using a 7 mm × 70-cm prosthetic graft (Advanta; Getinge, Gothenburg, Sweden), tunneled subcutaneously, and anastomosed in an end-to-side fashion to both CFAs.

**Fig. 2:**
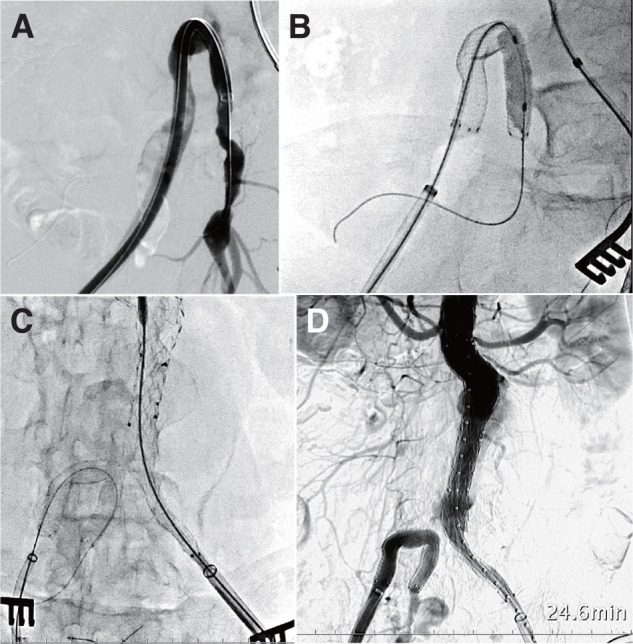
Intraoperative angiographic findings during endovascular aneurysm repair with internal iliac artery reconstruction. (**A**) Advancement of a 12-Fr Dry Seal sheath into the right internal iliac artery over a stiff guidewire. (**B**) Deployment of a 7 × 100-mm VBX balloon-expandable covered stent from the right external iliac artery to the right internal iliac artery to reestablish pelvic perfusion. (**C**) Deployment of the Endurant aorto-uni-iliac device with distal landing in the left external iliac artery after intentional occlusion of the left IIA. (**D**) Completion angiography demonstrating preserved right IIA perfusion, exclusion of the aneurysm sac, and absence of type I or type III endoleaks.

**Fig. 3:**
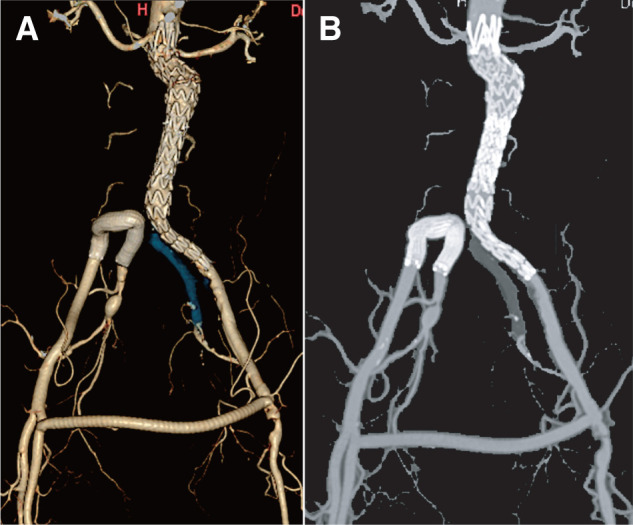
Postoperative computed tomography findings. (**A**) Three-dimensional computed tomography scan showing patency of the covered stent reconstruction and femorofemoral bypass with complete exclusion of the aneurysm sac. (**B**) Axial computed tomography image confirming patency of both the covered stent and femorofemoral bypass graft without evidence of endoleaks.

Completion angiography confirmed exclusion of the aneurysm, preservation of right IIA perfusion, and no evidence of type I or III endoleaks (**Fig. 2D**). The postoperative course was uneventful. Follow-up CT at 6, 12, and 21 months demonstrated the absence of aneurysm sac enlargement and sustained patency of both the covered stent reconstruction and femorofemoral bypass (**Fig. 3**).

## Discussion

Short CIA anatomy is a well-known challenge in EVAR, because it limits the distal sealing zone. In such cases, IIA embolization is frequently performed, but it may result in pelvic ischemic complications, including buttock claudication, sexual dysfunction, and colonic ischemia. Therefore, preservation of pelvic circulation is considered important. IBDs are the preferred option for maintaining IIA perfusion; however, their use is limited by anatomical constraints, particularly in cases with an extremely short and narrow CIA, as in the present case. Alternative techniques, including fenestration, chimney, and sandwich techniques, have been suggested; however, these approaches may be technically demanding and less reliable in patients with severe IIA stenosis or hypoplasia. In this context, we adopted a salvage hybrid strategy by combining covered stent-based IIA reconstruction with femorofemoral bypass. The covered stent served a dual purpose: preservation of the antegrade flow to the right IIA and intentional occlusion of the right CIA to eliminate inflow into the aneurysm sac. However, this configuration results in a nonanatomical, physiologically complex flow pattern that may theoretically increase the risk of thrombosis. In the present case, both internal iliac arteries exhibited severe ostial stenosis preoperatively, suggesting that baseline pelvic inflow was already reduced. Therefore, the additional hemodynamic impact of potential stent occlusion may be less pronounced compared with cases without pre-existing stenosis. Nevertheless, preservation of even limited antegrade pelvic perfusion may still contribute to maintaining collateral circulation and reducing the risk of ischemic complications. Thus, we believe that maintaining IIA flow, even in the presence of pre-existing stenosis, remains clinically meaningful in selected cases.

Furthermore, occlusion of the reconstructed right IIA would likely occur in a time-staggered manner relative to the already compromised contralateral side, which may allow for partial physiological adaptation and mitigate the risk of acute pelvic ischemia. In addition, this configuration may have a potential advantage in preventing retrograde perfusion from the IIA into the aneurysm sac, thereby reducing the risk of type II endoleak. Balloon-expandable covered stents have been reported as a feasible option for IIA revascularization with acceptable patency,^6)^ and devices such as the VBX (W. L. Gore & Associates) have demonstrated acceptable durability in iliac interventions, supporting their use in complex reconstructions.^7)^

An additional advantage of this approach was the ability to treat severe ostial stenosis of the right IIA (3.2 mm), achieving both luminal expansion and pelvic inflow preservation. However, we acknowledge that a small vessel caliber may increase the risk of thrombosis, necessitating careful patient selection and close postoperative surveillance.

The femorofemoral bypass restored perfusion to the right lower limb; however, this configuration inherently depends on a single inflow source and may represent a potential “single point of failure.” Therefore, the long-term patency of the bypass is critical, and continued follow-up is mandatory. In clinical practice, progressive graft stenosis or impending occlusion may be suspected based on a decline in the ankle–brachial index (ABI) and the development of intermittent claudication. Therefore, regular follow-up at approximately 6-month intervals is recommended. If graft occlusion is suspected, additional revascularization procedures, such as axillofemoral bypass, should be considered.

In the present case, no aneurysm sac enlargement was observed, and sustained patency of both the covered stent reconstruction and femorofemoral bypass was confirmed at 21 months, providing supportive mid-term evidence of feasibility. However, a longer follow-up period is required to establish long-term durability. This technique may be particularly useful in carefully selected patients with an extremely short CIA, severe IIA stenosis, and contraindications to open surgery, in whom the standard endovascular options are not feasible.

## Conclusion

Covered stent-based IIA reconstruction combined with femorofemoral bypass may represent a feasible alternative strategy to preserve pelvic circulation and ensure lower-limb perfusion in selected patients with a short CIA who are unsuitable for open repair.
